# Prognostic nomogram for elderly patients with acute respiratory failure receiving invasive mechanical ventilation: a nationwide population-based cohort study in Taiwan

**DOI:** 10.1038/s41598-020-70130-x

**Published:** 2020-08-04

**Authors:** Chun-Hsiang Hsu, Yao‐Min Hung, Kuo-An Chu, Chiu-Fan Chen, Chun-Hao Yin, Ching-Chih Lee

**Affiliations:** 1 Division of Chest Medicine, Kaohsiung Veterans General Hospital, Kaohsiung, Taiwan; 2 School of Medicine, National Yang Ming University, Taipei, Taiwan; 3 Department of Emergency Medicine, Kaohsiung Veterans General Hospital, Kaohsiung, Taiwan; 4 Yuhing Junior College of Health Care and Management, Kaohsiung, Taiwan; 5 Kaohsiung Municipal United Hospital, Kaohsiung, Taiwan; 6 Department of Nursing, Shu-Zen Junior College of Medicine and Management, Kaohsiung, Taiwan; 7 Department of Internal Medicine, Taitung Branch, Taipei Veterans General Hospital, Taitung, Taiwan; 8 Department of Medical Education and Research, Kaohsiung Veterans General Hospital, Kaohsiung, Taiwan; 9 Department of Otolaryngology, Head and Neck Surgery, Kaohsiung Veterans General Hospital, Kaohsiung, Taiwan; 10 Institute of Hospital and Health Care Administration, National Yang-Ming University, Taipei, Taiwan

**Keywords:** Outcomes research, Nomograms, Geriatrics

## Abstract

Patients in critical care medicine are ageing. There is limited literature evaluating long-term outcomes and prognostic factors for the growing number of elderly patients with acute respiratory failure (ARF) receiving invasive mechanical ventilation (IMV). Data on elderly patients (≧ 65 years old) with ARF receiving intubation and IMV during 2003–2012 were retrospectively collected from the national health database in Taiwan. We included 7,095 elderly patients. The 28-day mortality was 33%, the 60-day mortality was 47.5%, and the 1-year mortality was 70.4%. Patients were divided into groups: young-old (65–74 years), middle-old (75–84 years), and oldest-old (≧ 85 years). Patients in the oldest-old and middle-old groups had higher 1-year mortality than the young-old group (*p* < 0.001). The multivariate logistic regression revealed 9 significant factors associated with 1-year mortality, and these factors were used to develop a prognostic nomogram. The present study showed that the long-term prognosis of elderly patients with ARF and IMV is very poor. This nomogram can help physicians estimate the 1-year mortality of elderly patients in the early stage of ARF and assist in clinical decision making.

## Introduction

In most countries, the global trend of ageing is already taking place. The result is a rapid increase in the number of very old patients, with increased disability, heavy care burden, and higher medical costs^1–3^. A fast increase in the number of elderly patients in intensive care units (ICU) has been noted recently, especially for those age greater than 80 years old. The median age of ICU patients has increased to > 65 years old in many western countries^4^. The increasing percentage of very elderly and critical patients is challenging the effectiveness of critical care medicine. ICU treatment for very elderly critical patients may have a short-term survival benefit. However, the long-term mortality remained high, the 1-year mortality ranged from 40–70% in patients older than 80 years^4^.

Among elderly critical patients in ICU, invasive mechanical ventilation (IMV) is one of the most important factors predicting poor short-term (30 days) and long-term (1 year) outcomes^5^. Elderly patients already account for the majority of IMV patients. Literature shows that approximately 63–71% of all IMV patients were aged ≧ 65 years^6,7^. Previous literature reports a striking 1-year mortality rate of 72.5% in elderly critical patients ≧ 65 years old and receiving IMV^8^. Even for those who survived critical illness and were discharged, increase in disability and need for long-term care were serious issues. The high long-term mortality and substantial disability in elderly patients receiving IMV are usually poorer than the expectation of physicians and patient’s surrogates,therefore, aggressive treatment for this group of patients is controversial^8^.

Deciding intubation with IMV support for very elderly patients with acute respiratory failure (ARF) is a clinical dilemma, for both the patient and his surrogates. Predicting the outcomes of elderly patients with IMV is also a challenge for physicians, because there is no effective prognosis tool for these patients. Traditional predictive scores for critical patients tend to be less accurate when applied for very elderly patients^4^. Very few studies have evaluated factors associated with long-term outcome in elderly patients with IMV. A reliable prognosis tool predicting outcomes in critical elderly patients with IMV is required to assist clinical decision making.

The aim of this study was to evaluate the short-term and long-term (1-year) mortality rates of elderly patients with newly developed ARF receiving IMV using the National Health Insurance Research Database (NHIRD) of Taiwan. We also aimed to compare the outcome differences between each age group and to analyse the initial factors associated with 1-year mortality. A nomogram was developed for 1-year mortality rate prediction.

## Methods

### Study population

A retrospective observational study was conducted and the study data was obtained from the Longitudinal Health Insurance Database, a subset of the NHIRD. The National Health Insurance (NHI) programme in Taiwan is a single-payer system and has been implemented since 1995, approaching > 99% coverage of Taiwan’s population^9^. The NHIRD contains demographic data, disease diagnoses, prescriptions, procedures, and survival data. Personal information is encrypted and deidentified in this database. This study used the Longitudinal Health Insurance Database, which contains all the original claim data from a random sample of 1 million NHI beneficiaries. This study was approved by the Institutional Review Board of Kaohsiung Veterans General Hospital (IRB number: VGHKS15-EM10-02), which waived the requirement of informed consent. All the methods in this study were conducted in accordance with the directives of the Declaration of Helsinki.

From January 1, 2003, to December 31, 2012, we collected data on hospitalised elderly patients (≥ 65 years old) newly diagnosed with ARF (the International Classification of Diseases, Ninth Revision, Clinical Modification [ICD-9-CM] code: 518.81) and receiving endotracheal intubation with IMV. We excluded patients aged < 65 years and those admitted to local hospitals. For patients with multiple episodes of ARF receiving intubation and IMV, only the first episodes were chosen. All the enrolled patients were classified into three age groups: young-old, 65–74 years; middle-old, 75–84 years; and oldest-old, ≥ 85 years^10,11^.

### Measurement

The age, sex, hospital level, patient source (medical or surgical), main diseases, Charlson comorbidity index score (CCIS), inotropic agent use ≧ 3 days, ICU admission rate, ICU duration, IMV duration, 28-day mortality, 60-day mortality, and 1-year mortality data were collected. Main diseases associated with ARF were identified by the ICD-9-CM codes, including pneumonia (ICD-9-CM codes 480–487), obstructive lung disease (ICD-9-CM codes 490–496, which includes asthma, COPD, and bronchiectasis), heart failure (ICD-9-CM codes 428), sepsis (ICD-9-CM codes 038), neoplasm (ICD-9-CM codes 140–239), trauma (ICD-9-CM codes 800–959), cardiac arrest (ICD-9-CM codes 427.5), and acute kidney injury (ICD-9-CM codes 584). Inotropic agents included norepinephrine (ATC code C01CA03), dopamine (ATC code C01CA04), dobutamine (ATC code C01CA07), and epinephrine (ATC code C01CA24). We used CCIS as one variable in patients with single or multiple comorbidities^12^. Introduction of the CCIS calculation is presented in Supplementary Table S1.

The primary outcome was 1-year mortality. The secondary outcomes were factors associated with 1-year mortality, ICU durations, MV durations, 28-day and 60-day mortality. The first date of hospitalisation with ARF and MV was defined as the index date. The definition of mortality included marking of the NHI code as “died” or patient withdrawal from the NHI programme.

### Statistical analysis

Continuous variables (age, ICU duration and IMV duration) were evaluated for normal distribution using kurtosis and skewness. Normally distributed continuous variables were presented as mean ± standard deviation and were compared using one-way ANOVA. Non-normally distributed continuous variables were expressed as median and interquartile range (IQR) and were compared using Kruskal–Wallis test. Categorical variables were presented as number (percentage) and compared using the chi-squared test. The chi-square test was used to evaluate the 10-year linear trend of mortality rates. Univariate and multivariate logistic regression analyses were performed to identify the potential covariates associated with 1-year mortality. Statistical analyses were performed using SPSS version 20 (IBM SPSS, Armonk, NY, USA). A two-tailed *P* value < 0.05 was considered statistically significant.

Variables with statistical significance in multivariate logistic regression were selected for constructing the nomogram. A nomogram is a tool that provides graphical depictions of all variables in the model and enables the user to easily compute output probabilities^13^. For internal validation, we produced the calibration plots and evaluated the relationship between probabilities of predicted and observed 1-year mortality rates. The Hosmer–Lemeshow test was performed to evaluate the goodness-of-fit of the logistic regression model. The nomogram was built by using SAS software, version 9.4 (SAS Institute, Inc., Cary, NC).

## Results

### Clinical characteristics

**Figure 1:**
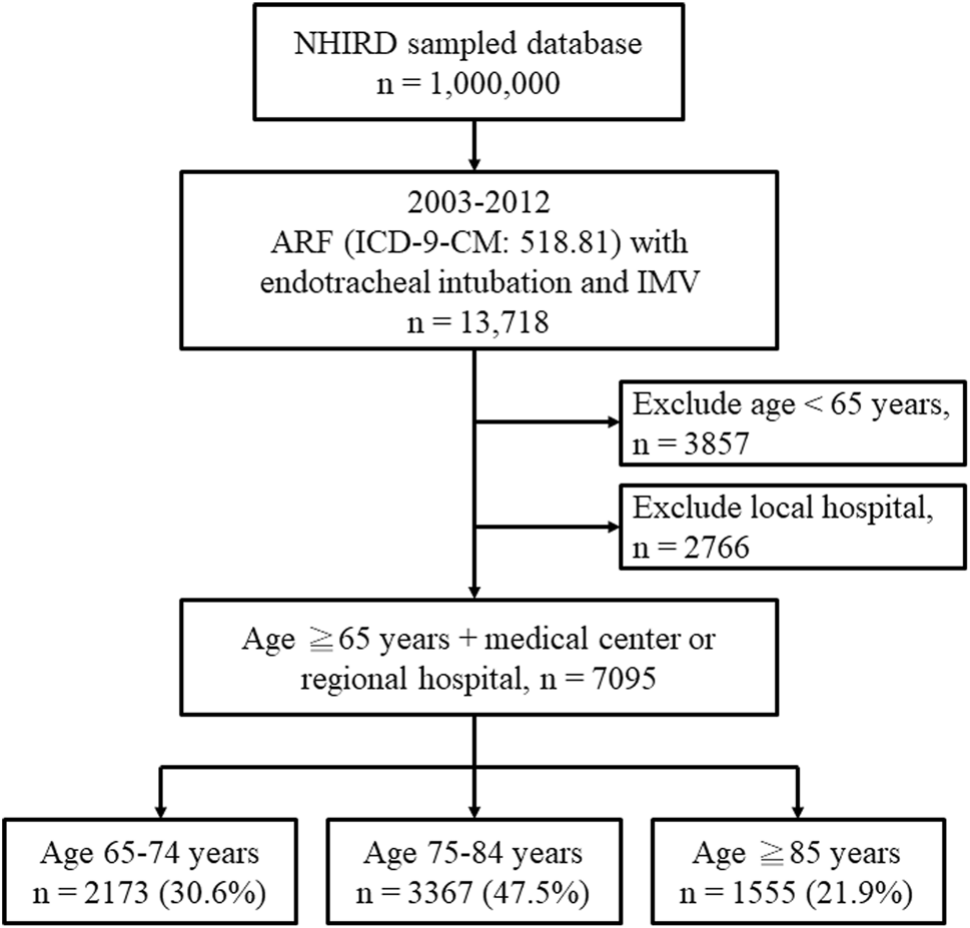
Flow chart of case collection. *ARF* acute respiratory failure, *IMV* invasive mechanical ventilation, *NHIRD* National Health Insurance Research Database.

During 2003–2012, a total of 13,718 patients with ARF receiving endotracheal intubation and IMV were identified. There were 3,857 patients aged < 65 years, and they were excluded. The remaining 9,861 patients aged ≧ 65 years, accounted for 71.9% of all the ARF and IMV population. Among these 9,861 elderly patients, 2,766 patients were admitted to a local hospital and were also excluded. Finally, 7,095 elderly patients were included in this study. The flow chart of patient collection is presented in Fig. 1. There were 2,173 patients in the young-old group (65–74 years, 30.6%), 3,367 in the middle-old group (75–84 years, 47.5%), and 1555 in the oldest-old group (≧ 85 years, 21.9%).

Table 1 presents the baseline characteristics of elderly patients with IMV. The median age was 79.2 (73–84) years. Overall, the majority of patients were male (60.1%), medical patients (85.5%), initially admitted to regional hospitals (61.9%), and with CCIS 0–2 (57.6%). Pneumonia and sepsis were the two most common main diseases associated with ARF. Patients in the older age groups were significantly associated with increased percentage of female, medical patients, and admission to regional hospitals. A higher percentage of patients with pneumonia and obstructive lung disease was observed in the older age groups. However, patients in the older age groups were associated with a lower percentage of neoplasm, acute kidney injury, and CCIS ≧ 3.

**Table 1:** Baseline characteristics of elderly patients with MV, n = 7,095.

Variables	65–74 years	75–84 years	≥ 85 years	*p* value
	n = 2,173	n = 3,367	n = 1555	
Age, median (IQR)	70.8 (68.1–73)	80 (77.6–82.3)	88 (86.4–90.9)	< 0.001
**Gender, n (%)**				0.001
Male	1,347 (62%)	2043 (61%)	873 (56%)	
Female	826 (38%)	1,324 (39%)	682 (44%)	
**Patient source, n (%)**				< 0.001
Medical	1818 (84%)	2,866 (85%)	1,380 (89%)	
Surgical	355 (16%)	501 (15%)	175 (11%)	
**Hospital level**^**a**^**, n (%)**				< 0.001
Medical center	946 (44%)	1,273 (38%)	481 (31%)	
Regional hospital	1,227 (56%)	2094 (62%)	1,074 (69%)	
**Main disease, n (%)**				
Pneumonia	744 (34%)	1,318 (39%)	725 (47%)	< 0.001
Obstructive lung disease	181 (8%)	374 (11%)	195 (13%)	< 0.001
Heart failure	152 (7%)	264 (8%)	121 (8%)	0.477
Sepsis	535 (25%)	832 (25%)	415 (27%)	0.270
Trauma	102 (5%)	152 (4%)	73 (5%)	0.937
Neoplasm	316 (14%)	337 (10%)	104 (7%)	< 0.001
Cardiac arrest	17 (1%)	27 (1%)	12 (1%)	0.993
Acute kidney injury	107 (5%)	130 (4%)	51 (3%)	0.031
Inotropics ≧ 3 days, n (%)	100 (5%)	171 (5%)	90 (6%)	0.267
**CCIS, n (%)**				< 0.001
0 ~ 2	1,185 (55%)	1924 (57%)	981 (63%)	
≥ 3	988 (45%)	1,443 (43%)	574 (37%)	
ICU admission, n (%)	2027 (97%)	3,135 (97%)	1,448 (97%)	0.967
ICU duration, median (IQR)	6 (2–13)	8 (3–15)	8 (3–15)	< 0.001
IMV duration, median (IQR)	6 (2–12)	7 (2–14)	7 (3–15)	< 0.001
28-day mortality, n (%)	729 (33%)	1,090 (32%)	524 (34%)	0.540
60-day mortality, n (%)	987 (45%)	1602 (48%)	780 (50%)	0.017
One-year mortality, n (%)	1,412 (65%)	2,382 (71%)	1,199 (77%)	< 0.001

^a^Hospital level of initial admission.

*SD* standard deviation, *CCIS* Charlson Comorbidity Index Score, *ICU* intensive care unit, *IMV* invasive mechanical ventilation, *IQR* interquartile range.

**Figure 2:**
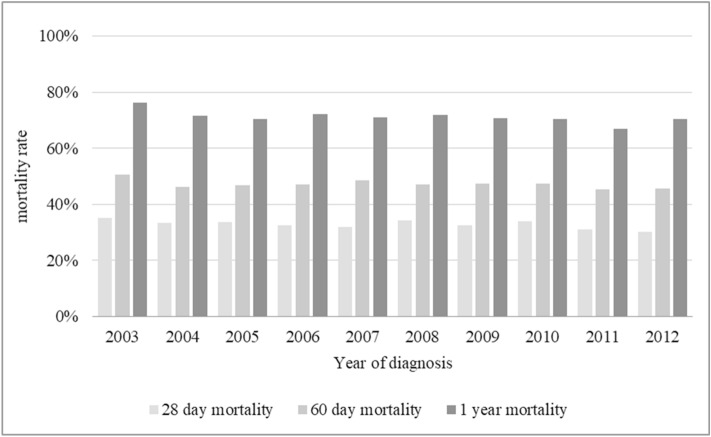
The 10-year trends of 28-day, 60-day, and 1-year mortality of elderly patients with acute respiratory failure receiving invasive mechanical ventilation. There were nonsignificant trends of decline in 28-day (*p* = 0.671), 60-day (*p* = 0.729), and 1-year mortality rate (*p* = 0.07).

The 28-day mortality rate was 33%, the 60-day mortality rate was 47.5% and the 1-year mortality rate was 70.4%. The 60-day mortality rate was higher in older age groups (45%, 48%, 50% with *p* value = 0.017). The 1-year mortality rate was also higher in older age groups (65%, 71%, 77% with *p* value < 0.001). However, the 28-day mortality was similar between the three age groups. Figure 2 showed the 10-year trends of 28-day, 60-day and 1-year mortality rate. The trends of decline in 28-day (*p* = 0.671), 60-day (*p* = 0.729), and 1-year mortality rate (*p* = 0.07) were not significant. Most of the patients were admitted to the ICU (97%), with median ICU duration of 7 (3–14) days and median IMV duration of 7 (2–13) days. Significant longer ICU durations (*p* < 0.001) and IMV durations (*p* < 0.001) were noted in older age groups. The ICU admission rate was similar between the three age groups.

### Factors associated with 1-year mortality

Table 2 shows the stepwise multivariate logistic regression analysis results for 1-year mortality. Significant factors associated with poorer outcome were middle-old (75–84 years, odds ratio [OR] = 1.44, 95% confidence interval [CI] = 1.28–1.63, *p* < 0.001), oldest-old ( ≧ 85 years, OR = 2.19, 95% CI = 1.87–2.55, *p* < 0.001), male (OR = 1.32, 95% CI = 1.19–1.48, *p* < 0.001), sepsis (OR = 1.85, 95% CI = 1.61–2.11, *p* < 0.001), neoplasm (OR = 4.10, 95% CI = 3.20–5.25, *p* < 0.001), cardiac arrest (OR = 5.10, 95% CI = 2.01–12.91, *p* < 0.001), and CCIS ≥ 3 (OR = 1.61, 95% CI = 1.44–1.79, *p* < 0.001). Significant factors associated with better outcomes were obstructive lung disease (OR = 0.62, 95% CI = 0.53–0.73, *p* < 0.001), heart failure (OR = 0.82, 95% CI = 0.68–0.99, *p* = 0.042), and trauma (OR = 0.73, 95% CI = 0.58–0.93, *p* = 0.009).

**Table 2:** Stepwise multivariate logistic regression analysis for 1-year mortality in elderly patients with MV.

Variables	Beta	adjusted OR (95% CI)	*p* value
**Age group**			
65 ~ 74		1	
75 ~ 84	0.366	1.44 (1.28–1.63)	< 0.001
≥ 85	0.782	2.19 (1.87–2.55)	< 0.001
**Gender**			
Male	0.280	1.32 (1.19–1.48)	< 0.001
Female		1	
**Obstructive lung disease**			
Yes	− 0.474	0.62 (0.53–0.73)	< 0.001
No		1	
**Heart failure**			
Yes	− 0.198	0.82 (0.68–0.99)	0.042
No		1	
**Sepsis**			
Yes	0.613	1.85 (1.61–2.11)	< 0.001
No		1	
**Trauma**			
Yes	− 0.313	0.73 (0.58–0.93)	0.009
No		1	
**Neoplasm**			
Yes	1.410	4.10 (3.20–5.25)	< 0.001
No		1	
**Cardiac arrest**			
Yes	1.629	5.10 (2.01–12.91)	< 0.001
No		1	
**CCIS_group**			
0 ~ 2		1	
≥ 3	0.474	1.61 (1.44–1.79)	< 0.001

*CCIS* Charlson Comorbidity Index Score, *CI* confidence interval, *MV* mechanical ventilation.

### Nomogram for 1-year mortality estimation

**Figure 3:**
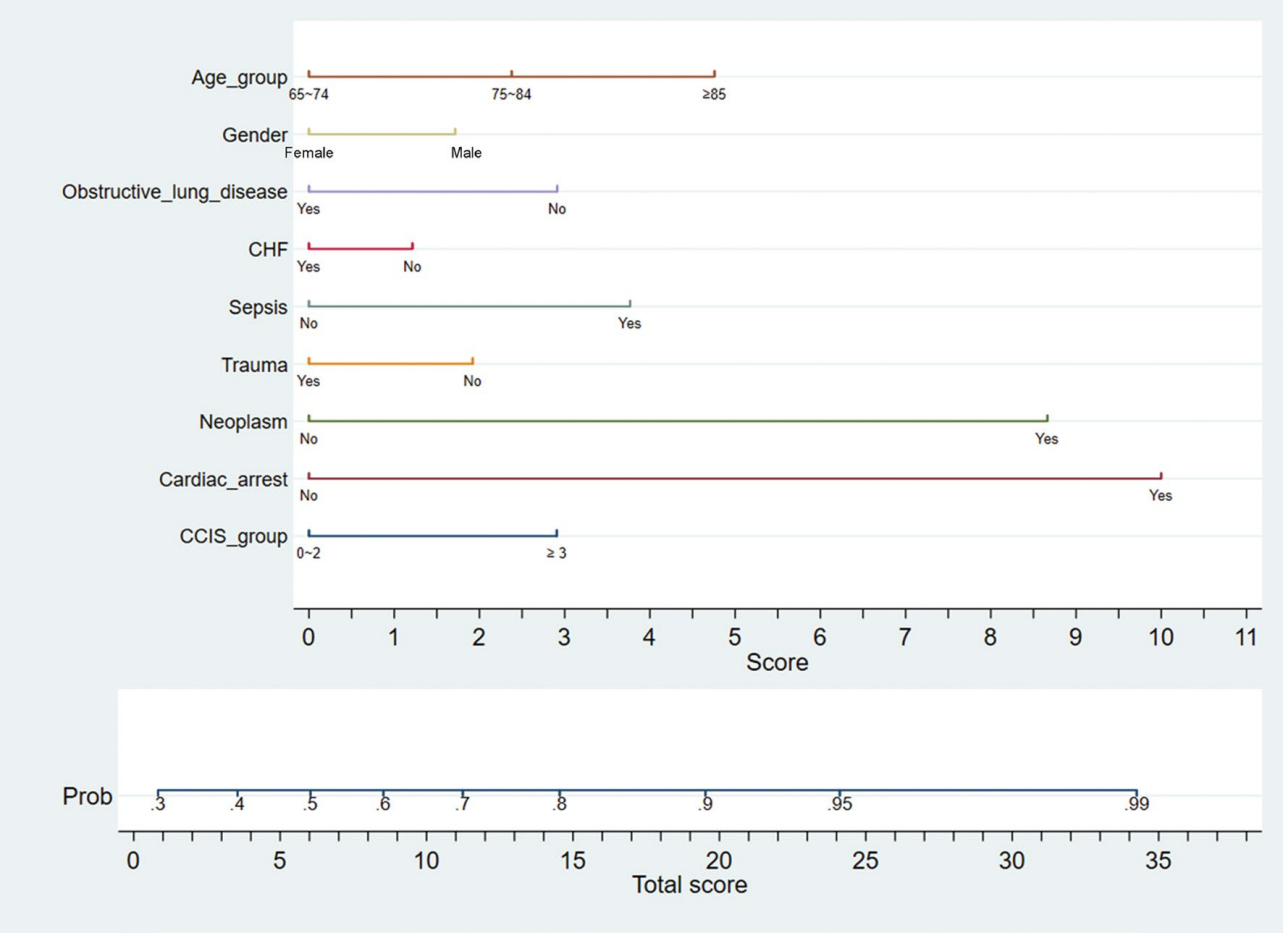
Nomogram for the prediction of 1-year mortality in elderly patients with invasive mechanical ventilation. A nomogram was developed according to 9 significant factors associated with 1-year mortality in the stepwise multivariate logistic regression. Obstructive lung disease included COPD, asthma, and bronchiectasis. Neoplasm indicated active cancer. *ARF* acute respiratory failure, *CCIS* Charlson comorbidity index score, *COPD* chronic obstructive lung disease, *IMV* invasive mechanical ventilation.

**Figure 4:**
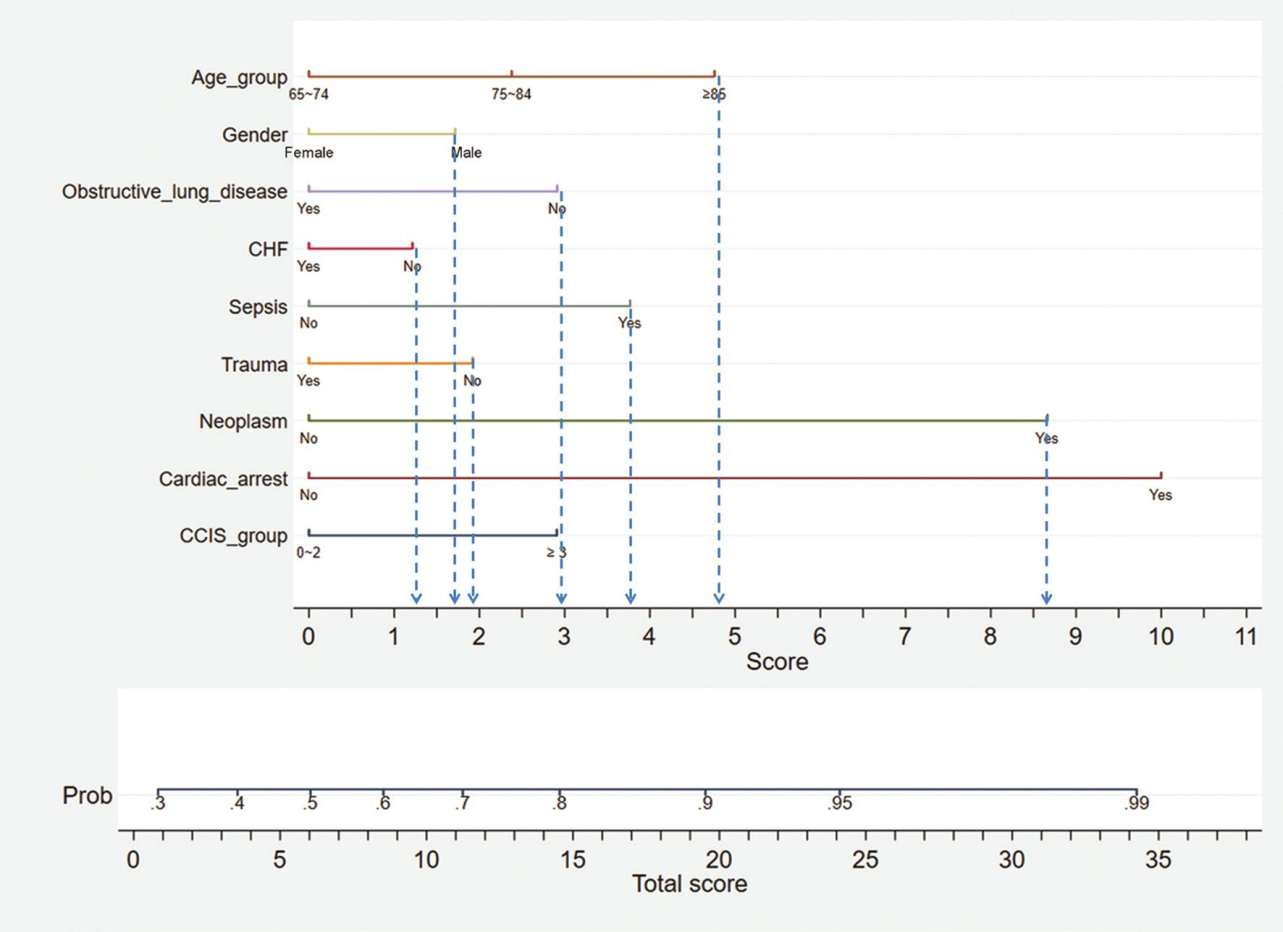
Nomogram application example. A straight line was drawn downward to determine the points for the variables (blue dashed arrow). For a male (1.7 points) patient with ARF, aged 85 years (4.8 points), with sepsis (3.8 points) and lung cancer (8.7 points), with CCIS* = 2 (0 points) and no obstructive lung disease (3 points), no heart failure (1.2 points), and no trauma (2 points), the total score will be 25.2. The probability of 1-year mortality will be 95% according to the total score axis. In this example case, CCIS = 2 owing to the lung cancer (assume no distant metastasis). *ARF* acute respiratory failure, *CCIS* Charlson comorbidity index score.

**Figure 5:**
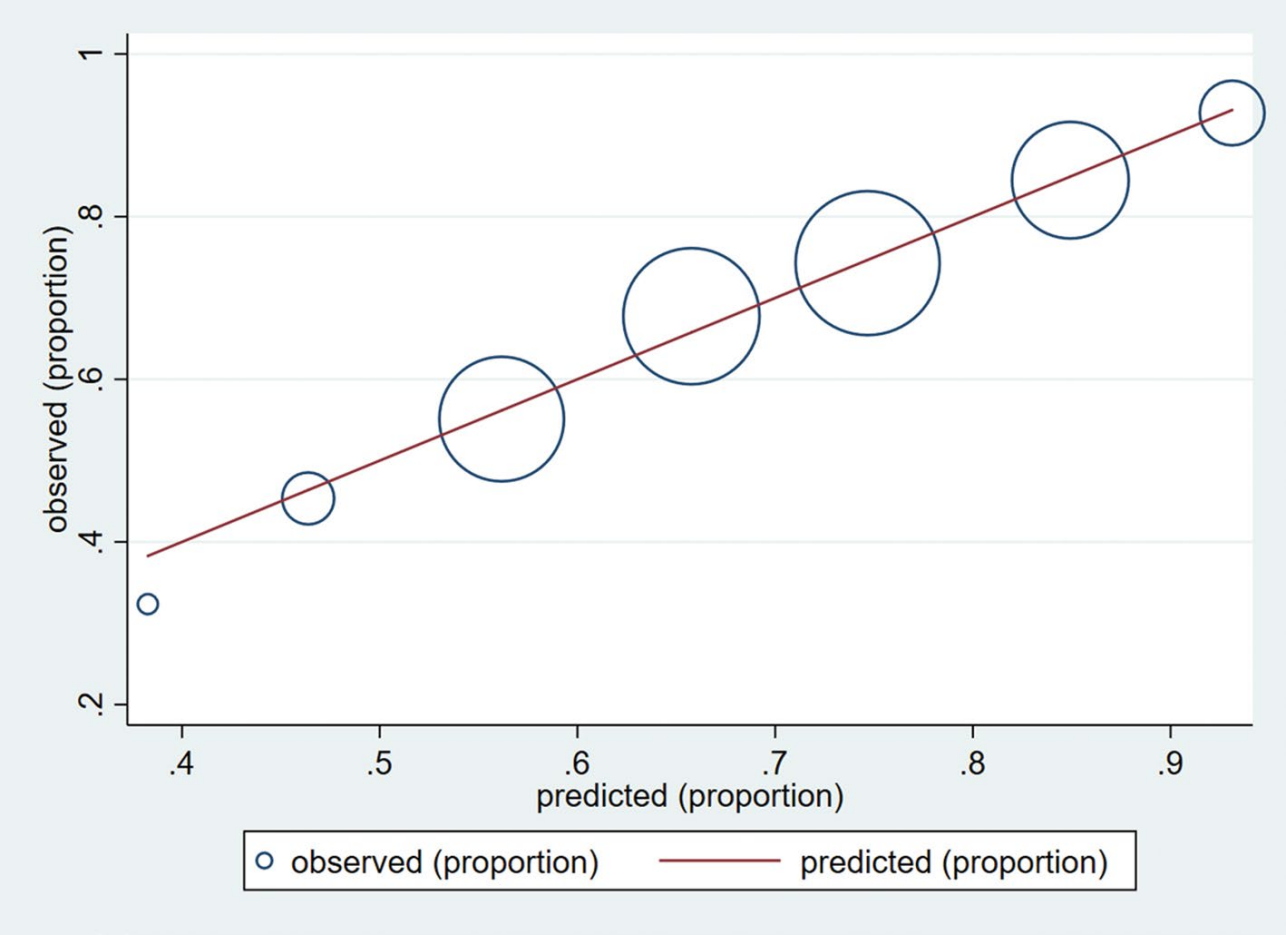
Calibration plot for the comparison of predictive and observed 1-year mortality in elderly patients with invasive mechanical ventilation. The diagonal line represents the ideal reference line where predicted probabilities would match the observed probabilities. The blue circle represents the performance of the nomogram, of which a closer fit to the diagonal line represents a better prediction. The size of the blue circle is proportional to the case number of observed mortality rate. The Hosmer–Lemeshow goodness-of-fit test showed *p* = 0.686, indicating no significant difference between predicted and observed probabilities.

A nomogram was constructed by using 9 significant factors (categorised age, sex, obstructive lung disease, CHF, sepsis, trauma, neoplasm, cardiac arrest, and categorised CCIS) identified in multivariate logistic regression analysis (Fig. 3). For example, in a 85-year-old male patient with sepsis, lung cancer, and CCIS = 2, the estimated 1-year mortality for ARF with IMV will be 95% (Fig. 4). Internal validation was performed by using the entire sample to produce a calibration plot for comparison of predicted and observed probabilities of 1-year mortality and demonstrated good agreement (Fig. 5). In the calibration plot, the size of blue circle is proportional to the case number of observed mortality rate. The Hosmer–Lemeshow test for goodness-of-fit yielded a nonsignificant difference (*p* = 0.686) between predicted and observed probabilities, indicating that this predictive tool is effective and reliable.

## Discussion

To the best of our knowledge, this is the first study to evaluate the long-term outcomes for ARF with intubation and IMV in young-old, middle-old and oldest-old patients. Despite recent advances in critical care medicine, the long-term mortality in the elderly critical patients is still very high. This study showed that the mean 1-year mortality of elderly critical patients with IMV is as high as 70%, and the outcome is poorer in the oldest-old group. In the prognostic factor analysis, cardiac arrest, neoplasm, and older age are the three most important factors determining the 1-year mortality. We developed a nomogram by using 9 baseline clinical factors to predict 1-year mortality of elderly patients with IMV. This tool helps the physicians to estimate the 1-year mortality of elderly patients during the early course of ARF and assists clinical decision making.

Elderly patients already account for the majority of IMV patients. A previous NHI study in Taiwan, from 2004 to 2008, showed that up to 71.1% of all IMV patients were aged ≧ 65 years and 46.6% were aged ≧ 75 years^6^. Another study in Israel showed that 63.7% of all patients with IMV were aged ≧ 65 years^7^. These findings are similar to ours, with 71.9% of the entire population with ARF and IMV aged ≧ 65 years. Our study also showed that elderly patients receiving IMV were older than we expected, with most patients (47.5%) in the 75–84 years age group, and patients in the ≧ 85 years age group were also common (21.9%). This showed that elderly patients with IMV were ageing than that reported previously, of which 40.2% patients were in the 65–74 years, 45.6% in the 75–84 years, and 14.2% in the ≧ 85 years age groups^7^.

Although critical patients keep ageing, many of them still choose aggressive treatment with intubation and IMV support. However, the efficacy and treatment outcomes of these intensive and invasive procedures are controversial. Among the three age groups, the oldest age group was associated with the lowest percentage of patients with CCIS ≧ 3. The possible explanation is that for elderly patients with few comorbidities, better prognosis can be expected, and a decision of intubation with IMV is more likely to be made in an ARF situation. In contrast, for elderly patients with multiple comorbidities, a decision of not receiving IMV is more likely to be made by patients or their surrogates, and this would be more prominent in the oldest age group.

Recent literature evaluating nonselective adult patients with ARF and IMV showed in-hospital mortality of 26–36% and 28-day mortality of 30–33%^14–16^. In present study, the 28-day mortality of 33% is concordant with the short-term outcomes described in above literature. As for the long-term mortality, previous literature evaluating nonselective elderly patients ≧ 65 years old with ARF and IMV showed a 1-year mortality of 72.5%^8^. Another study evaluating IMV patients showed a 1-year mortality of 69% in men aged 60–74 years and a 1-year mortality of 77% in those aged ≧ 75 years^17^. In a study of elderly stroke patients (age > 65 years) with ARF and IMV, the 6-month mortality was 60%^18^. In a recent study of a COPD patient with long-term oxygen treatment, receiving IMV owing to ARF, the 1-year mortality was as high as 68.5%^19^. The poor long-term prognosis in above studies is compatible to our result of 70.4% 1-year mortality. For elderly critical patients with ARF, intensive treatment with intubation and IMV might be beneficial for short-term outcomes,however, the 1-year outcome remains quite poor. In addition, critical illness resulted in considerable disability progression, and only few patients had good recovery^20^. As a result, very few elderly patients with IMV could survive for 1 year and retained a good functional status.

This study showed that cardiac arrest and neoplasm were the two strongest factors associated with 1-year mortality in elderly patients with IMV; this is consistent with that reported previously. In a study in 1993 in the US, cardiac arrest was associated with the worst long-term prognosis, when compared to other causes of ARF with IMV (1-year mortality: 85% vs. 56 ~ 77%)^17^. Other studies also showed that cancer patients with ARF and IMV had very poor prognosis, with a hospital mortality rate of approximately 62–82% and 6-month mortality rate of approximately 77–97%^21–23^. Older age is another important factor associated with poor prognosis in our study. Patients in the oldest-old (≧ 85 years) and middle-old (75–84 years) groups had significant higher 1-year mortality than the young-old (65–74 years) group (*p* < 0.001). Previous studies showed a similar trend of poor prognosis in elderly patients, but the evaluated age was younger (> 65 and > 70 years) ^17,24^.

This study also showed that obstructive lung disease (including asthma, COPD and bronchiectasis), heart failure, and trauma were associated with better long-term outcomes, and this trend is similar to that reported in a study evaluating short-term outcomes in adult patients with IMV^24^. The reason for better prognosis in the above three groups may be explained by that patients with these diseases were prone to developing ARF but were also more likely improved after intensive treatment, when compared to other diseases.

With the increasing population of elderly patients, the need for critical care and IMV support will increase rapidly, and critical care system overloading will become a serious problem. In fact, literature has shown that elderly critical patients had a greater rejection rate (18–36%) for ICU admission, when compared to the rejection rate (11–15%) of those aged < 65 years^25^. In some areas, a significant percentage (7.6–38.4%) of critical patients with IMV even had to stay in the general ward owing to shortage of ICU beds, and the condition was more severe for elderly patients (up to 51.1%)^7,26,27^. In the near future, many hospitals will be facing similar problems, when the increasing number of elderly patients with IMV exceeds the reserve of critical care manpower and resources.

On the other hand, elderly critical patients with IMV already approached a poor prognosis that is similar to terminal illness, which is most commonly defined as an illness with life expectancy of less than 6 months^28^. For patients with a very high predicted 1-year mortality, ineffective medical treatment may result in heavy medical costs and long-term care burden. Therefore, palliative care without IMV support in the beginning of ARF or IMV withdrawal after a short period of intensive treatment trial, may be a possible alternative choice for elderly patients with ARF.

A nomogram is a graphical representation of multivariable model for prognosis prediction and is increasingly been used in oncology. It helps physicians predict patient’s individual probability of events, such as mortality, by integrating multiple clinical factors in a visualised and straightforward manner^29^. The use of nomogram in respiratory medicine is still less,nomograms for the estimation of prognosis of resected non-small cell lung cancer, prognosis of small cell lung cancer, diagnostic accuracy of asthma, in-hospital mortality of asthma exacerbation, and success rate of planned extubation have been developed^30–34^.

This study developed the first nomogram to estimate the 1-year mortality in elderly patients with ARF and IMV, by using multiple simple baseline clinical factors. This nomogram can help physicians predict 1-year mortality of individual patients in the early stage of ARF, even before deciding to intubate. This prognosis information can help physicians and patient’s surrogates with decision making between aggressive and palliative treatment.

This study has several limitations. Firstly, the NHIRD database did not include the data of laboratory exams, vital signs and the clinical parameters. Therefore, the severity score was unavailable. Secondly, we did not evaluate the length of hospital stay, short-term and long-term weaning outcomes. Thirdly, we did not evaluate the premorbid functional status and frailty, the important factors predicting short-term and long-term mortality in critically ill patients^5,35,36^. The functional status is not available in the NHIRD database. Fourthly, the postoperative patients with simple and rapid weaning were not included in this study. Therefore, our results were not applicable to this patient group. We also could not differentiate elective surgical patients from emergency surgical patients. Lastly, health care in Taiwan is cheaper and more available than in many countries. The number of ICU beds in Taiwan^37^ (31.7 per 100,000 persons) is similar to that in the United States^38^ (34.7 per 100,000 persons), and is higher than that in most of the European countries^39^ (range from 4.2 to 29.2 per 100,000 persons). In countries with high medical costs or limited medical resources, the patient’s attitude towards critical care may be different. Therefore, our results may not be generalisable in other countries. The strengths of this study are that this is a nationwide population-based database study, the diagnosis of ARF and IMV is very reliable, and the long-term outcome is also complete.

With global ageing, more elderly critical patients with ARF will be admitted to hospital, and the tough decision whether to intubate with IMV support has to be made. This study showed that the 1-year mortality of elderly patients with ARF receiving IMV is very high, and the effectiveness of IMV treatment may be controversial. However, patients with ARF due to obstructive lung diseases, heart failure, and trauma were associated with better 1-year prognoses in our study, suggesting that IMV is more effective in these patient groups. The nomogram developed in this study enables physicians to estimate the 1-year mortality in the early stage of ARF and assist clinical decision making between aggressive and palliative treatment. However, the weaning outcome and effectiveness of non-invasive ventilation for elderly patients with ARF requiring IMV are also important issues to be investigated. Future research focusing on these topics is necessary to provide more comprehensive outcome information for elderly patients with ARF.

## Supplementary information

Supplementary file1
