# Supplementary material for: Prognostic nomogram for elderly patients with acute respiratory failure receiving invasive mechanical ventilation: a nationwide population-based cohort study in Taiwan
Source: Sci Rep. 2020 Aug 4;10:13161. doi: 10.1038/s41598-020-70130-x (PMC7403322; doi:10.1038/s41598-020-70130-x)
Supplement: Supplementary file 1 — Supplementary file1 [file 41598_2020_70130_MOESM1_ESM.docx]

**Prognostic Nomogram for Elderly Patients with Acute Respiratory Failure Receiving Invasive Mechanical Ventilation: A Nationwide Population-Based Cohort Study in Taiwan**

Chun-Hsiang Hsu, Yao‐Min Hung, Kuo-An Chu, Chiu-Fan Chen, Chun-Hao Yin, Ching-Chih Lee

**Supplementary file**

Table S1. Introduction for Charson Comorbidity Index Score calculation.

| Comorbidity | score |
| --- | --- |
| Myocardial infarct | 1 |
| Congestive heart failure | 1 |
| Peripheral vascular disease | 1 |
| Cerebrovascular disease | 1 |
| Dementia | 1 |
| Chronic pulmonary disease | 1 |
| Connective tissue disease | 1 |
| Ulcer disease | 1 |
| Mild liver disease | 1 |
| Diabetes | 1 |
| Diabetes with end organ damage | 2 |
| Hemiplegia | 2 |
| Moderate to severe renal disease | 2 |
| Any tumor | 2 |
| Leukemia or lymphoma | 2 |
| Moderate to severe liver disease | 3 |
| Metastatic solid tumor | 6 |
| AIDS | 6 |

Reproduced from Charson et al. 1987^12^

Charson Comorbidity Index Score = the sum score of the comorbidities.

AIDS = acquired immune deficiency syndrome.
